# Supplementary material for: Structure of alpha-synuclein fibrils derived from human Lewy body dementia tissue
Source: Nat Commun. 2024 Mar 29;15:2750. doi: 10.1038/s41467-024-46832-5 (PMC10980826; doi:10.1038/s41467-024-46832-5)
Supplement: Supplementary file 2 — Reporting Summary [file 41467_2024_46832_MOESM2_ESM.pdf]

nature portfolio

Corresponding author(s): Paul Kotzbauer, Chad Rienstra

Last updated by author(s): Feb 14, 2024

Reporting Summary

Nature Portfolio wishes to improve the reproducibility of the work that we publish. This form provides structure for consistency and transparency in reporting. For further information on Nature Portfolio policies, see our Editorial Policies and the Editorial Policy Checklist.

Statistics

For all statistical analyses, confirm that the following items are present in the figure legend, table legend, main text, or Methods section.

n/a	Confirmed	
☐	☒	The exact sample size (n) for each experimental group/condition, given as a discrete number and unit of measurement
☐	☒	A statement on whether measurements were taken from distinct samples or whether the same sample was measured repeatedly
☐	☒	The statistical test(s) used AND whether they are one- or two-sided<br>*Only common tests should be described solely by name; describe more complex techniques in the Methods section.*
☒	☐	A description of all covariates tested
☐	☒	A description of any assumptions or corrections, such as tests of normality and adjustment for multiple comparisons
☐	☒	A full description of the statistical parameters including central tendency (e.g. means) or other basic estimates (e.g. regression coefficient) AND variation (e.g. standard deviation) or associated estimates of uncertainty (e.g. confidence intervals)
☐	☒	For null hypothesis testing, the test statistic (e.g. F, t, r) with confidence intervals, effect sizes, degrees of freedom and P value noted<br>*Give P values as exact values whenever suitable.*
☒	☐	For Bayesian analysis, information on the choice of priors and Markov chain Monte Carlo settings
☒	☐	For hierarchical and complex designs, identification of the appropriate level for tests and full reporting of outcomes
☒	☐	Estimates of effect sizes (e.g. Cohen's d, Pearson's r), indicating how they were calculated

Our web collection on statistics for biologists contains articles on many of the points above.

Software and code

Policy information about availability of computer code

Data collection	EPU (ThermoFisher Scientific) - Cryo-EM data collection<br>NMRbox<br>Metamorph 6.1
Data analysis	MOTIONCOR2 1.5<br>GCTF 1.06<br>cryoSPARC v4.0<br>RELION 3.1<br>EMAN2<br>MpUL-multi<br>NMRPipe<br>SPARKY 3<br>XPLOR-NIH<br>NAMD 3.0<br>Graphpad Prism 10

For manuscripts utilizing custom algorithms or software that are central to the research but not yet described in published literature, software must be made available to editors and reviewers. We strongly encourage code deposition in a community repository (e.g. GitHub). See the Nature Portfolio guidelines for submitting code & software for further information.

Data

Policy information about availability of data

All manuscripts must include a data availability statement. This statement should provide the following information, where applicable:

- Accession codes, unique identifiers, or web links for publicly available datasets
- A description of any restrictions on data availability
- For clinical datasets or third party data, please ensure that the statement adheres to our policy

The datasets generated during and/or analysed during the current study are available from the corresponding author on reasonable request. The ten lowest energy structural models have been deposited in the Protein Data Bank (PDB) under accession number 8FPT [https://doi.org/10.2210/pdb8FPT/pdb]. NMR data have been deposited in the Biological Magnetic Resonance Bank: BMRB 31068 [https://doi.org/10.13018/BMR31068] (Structure of Alpha-Synuclein fibrils derived from Human Lewy Body Dementia Tissue). Molecular dynamics simulation scripts and data have been submitted in the DRYAD public repository, [10.5061/dryad.tx95x6b4z]. Backbone assignments are provided in BMRB ID 51678 [https://doi.org/10.13018/BMR51678]. Chemical shifts assignments have been submitted to the BMRbig, under the entry ID: BMRbig83. Additional source data is included in source data file.

Research involving human participants, their data, or biological material

Policy information about studies with human participants or human data. See also policy information about sex, gender (identity/presentation), and sexual orientation and race, ethnicity and racism.

Reporting on sex and gender	Findings do not apply to any particular sex. Sex and gender were not considered in the study design. Sex was determined based on self-reporting. Disaggregated sex data is provided and information has been collected by self-reporting. Individual level data has been collected. LBD: 5 Male & 2 Female, MSA: 3 Male & 1 Female, Disease-control: 2 Male & 1 Female.
Reporting on race, ethnicity, or other socially relevant groupings	The study did not use any race, ethnicity or any other socially relevant groupings.
Population characteristics	See supplementary table 10. Disaggregated sex data is provided, primary diagnosis by the neuropathology is also listed. Diagnosis: 7 cases: LBD, 4 cases: MSA, 3 cases: disease-control
Recruitment	Samples were selected based on neuropathological examination and brain tissue availability, which is unlikely to have impacted the results.
Ethics oversight	The Movement Disorders Brain Bank, Washington University, St. Louis, MO, provided clinically and neuropathologically well-characterized postmortem frozen brain tissue. The Human Research Protection Office at Washington University in Saint Louis approved this study. Written informed consent to perform a brain autopsy was obtained from all participants. After death, the immediate next-of-kin were contacted and confirmed consent for brain removal and retention of brain tissue for research purposes.

Note that full information on the approval of the study protocol must also be provided in the manuscript.

Field-specific reporting

Please select the one below that is the best fit for your research. If you are not sure, read the appropriate sections before making your selection.

☒ Life sciences ☐ Behavioural & social sciences ☐ Ecological, evolutionary & environmental sciences

For a reference copy of the document with all sections, see nature.com/documents/nr-reporting-summary-flat.pdf

Life sciences study design

All studies must disclose on these points even when the disclosure is negative.

Sample size	Sample sizes were chosen based on brain tissue availability and practical considerations regarding the amount of amplified fibrils required for collection of SSNMR data.<br>We have analyzed SSNMR data for three independently prepared amplified fibril samples (produced with three different isotopic labeling strategies) derived from the same case (LBD1). We also analyzed 2D spectra from 2 LBD cases and 1D spectra from three LBD cases.<br>Figure 2: Three cases of LBD and three cases of MSA.<br>Figure 7: Three cases of LBD.<br>Figure 8: Four cases of LBD and four cases of MSA.<br>Supplementary Fig 1-5: Amplified fibrils from three LBD, MSA and Control cases.<br>Supplementary Fig. 7: Amplified fibrils from five LBD cases after 6 cycles and amplified fibrils from three cases of LBD after 4 and 2 cycles.<br>Supplementary Fig. 8: Amplified fibrils from three cases of MSA and control after 6 cycles.<br>Supplementary Fig. 9: Amplified fibrils from three cases of LBD, MSA and control after 2 and 4 cycles. Amplified fibrils from five cases of LBD, four cases of MSA and three cases of control after 6 cycles.<br>Supplementary Fig. 10: Amplified fibrils from two LBD cases.<br>Supplementary Table 5 and 6: Three samples of LBD amplified fibrils after 6 cycles.<br>Supplementary Table 7: Three samples of LBD and MSA amplified fibrils after 6 cycles.

Data exclusions	There were no data exclusions.
Replication	All attempts at replication were successful for experiments described below:<br>Fig. 1: Similar results were obtained from negative stain TEM images collected for at least three independently prepared fibril samples.<br>Fig. 2: Similar results were observed in more than three independent experiments examining amplified fibrils derived from three cases of LBD and three cases of MSA.<br>Fig. 7: We have analyzed SSNMR data for three independently prepared amplified fibril samples (produced with three different isotopic labeling strategies) derived from the same case (LBD1). We also analyzed 2D spectra from 2 cases and 1D spectra from three LBD cases.<br>Fig. 8: Similar results were obtained from two independent experiments from amplified fibrils derived from caudate samples for four cases of LBD and four cases of MSA.<br>Supplementary Fig. 1 to 5: A minimum of 10 micrographs were collected for each sample. Similar results were obtained from at least three independently prepared fibril samples.<br>Supplementary Fig. 6: Similar results were obtained from at least three independently prepared fibril samples.<br>Supplementary Fig. 7: A minimum of 10 micrographs were analyzed for diameter measurements for each sample. Amplified fibrils from five cases of LBD after 6th cycle, Amplified fibrils from three cases of LBD after 4th and 2nd cycle.<br>Supplementary Fig. 8: A minimum of 10 micrographs were analyzed for diameter measurements for each sample. Amplified fibrils from five cases of MSA and Control after 6th cycle.<br>Supplementary Fig. 9: Similar results were obtained from two independent experiments.<br>Supplementary Fig. 10: Experiment was done once.<br>Supplementary Table 7: Similar results were obtained from two independent experiments.
Randomization	Randomization is not relevant for this study. Groups were defined by neuropathologic diagnosis of autopsy cases for the data in Figures 2 and 8.
Blinding	Investigators were blinded to group allocation during data collection and data analysis for Supplementary Fig. 7, 8 and Supplementary Table 7. Blinding was not performed for other data as the perceived risk of detection/performance bias was deemed negligible.

Reporting for specific materials, systems and methods

We require information from authors about some types of materials, experimental systems and methods used in many studies. Here, indicate whether each material, system or method listed is relevant to your study. If you are not sure if a list item applies to your research, read the appropriate section before selecting a response.

Materials & experimental systems

n/a	Involved in the study	
☐	☒	Antibodies
☐	☒	Eukaryotic cell lines
☒	☐	Palaeontology and archaeology
☒	☐	Animals and other organisms
☒	☐	Clinical data
☒	☐	Dual use research of concern
☒	☐	Plants

Methods

n/a	Involved in the study	
☒	☐	ChIP-seq
☒	☐	Flow cytometry
☒	☐	MRI-based neuroimaging

Antibodies

Antibodies used	Syn303, Biolegend, Catalog 824301, RRID AB_2564879. Used at final concentration of 1ug/ml.<br>Syn1: BD Transduction Laboratories, Catalog# 610787, Clone 42/alpha-synuclein, RRID: AB_398107. Used at final concentration of 1ug/ml.<br>Syn211: Invitrogen, Catalog# 32-8100, RRID: AB_2533094. Used at final concentration of 1ug/ml.<br>13G5: In-house antibody. Used at final concentration of 1ug/ml.<br>Anti-mouse IgG, HRP-linked Antibody: Cell Signaling, Catalog: 7076, Used at 1:5000 dilution.
Validation	The western blot data shown in Supplementary Figure 10 included a lane with full-length recombinant alpha-synuclein protein.<br>For antibodies Syn303, Syn1, Syn211, HRP-Anti-mouse, relevant cited publications are available on respective manufacturers website.<br>For in-house antibody 13G5, see PMID 35060360 and PMID 32024799 for publications.

Eukaryotic cell lines

Policy information about cell lines and Sex and Gender in Research

Cell line source(s)	We utilized the previously described HEK293T "biosensor" cell line stably expressing alpha-synuclein (A53T)-CFP/YFP fusion proteins (Yamasaki, T.R. et al. Parkinson's disease and multiple system atrophy have distinct alpha-synuclein seed characteristics. J Biol Chem 294, 1045-1058 (2019)).

Authentication	We authenticated the biosensor cell line by verifying that appropriate levels of inclusion were formed after seeding this cell line with an in vitro assembled alpha-synuclein fibril preparation.
Mycoplasma contamination	The cell line was not tested for mycoplasma contamination.
Commonly misidentified lines (See ICLAC register)	HEK293T is not on the list of commonly misidentified lines.

Plants

Seed stocks	*Report on the source of all seed stocks or other plant material used. If applicable, state the seed stock centre and catalogue number. If plant specimens were collected from the field, describe the collection location, date and sampling procedures.*
Novel plant genotypes	*Describe the methods by which all novel plant genotypes were produced. This includes those generated by transgenic approaches, gene editing, chemical/radiation-based mutagenesis and hybridization. For transgenic lines, describe the transformation method, the number of independent lines analyzed and the generation upon which experiments were performed. For gene-edited lines, describe the editor used, the endogenous sequence targeted for editing, the targeting guide RNA sequence (if applicable) and how the editor was applied.*
Authentication	*Describe any authentication procedures for each seed stock used or novel genotype generated. Describe any experiments used to assess the effect of a mutation and, where applicable, how potential secondary effects (e.g. second site T-DNA insertions, mosiacism, off-target gene editing) were examined.*
